# Autistic Heterogeneity: Linking Uncertainties and Indeterminacies

**DOI:** 10.1080/09505431.2016.1238886

**Published:** 2016-10-31

**Authors:** Gregory Hollin

**Affiliations:** ^a^ School of Sociology and Social Policy, University of Leeds, UK

**Keywords:** autism, neuroscience, psychology, uncertainty work, indeterminacy work

## Abstract

Autism is a highly uncertain entity and little is said about it with any degree of certainty. Scientists must, and do, work through these uncertainties in the course of their work. Scientists explain uncertainty in autism research through discussion of *epistemological uncertainties* which suggest that diverse methods and techniques make results hard to reconcile, *ontological uncertainties* which suggest doubt over taxonomic coherence, but also through reference to autism’s *indeterminacy* which suggests that the condition is inherently heterogeneous. Indeed, indeterminacy takes two forms—an inter-personal form which suggests that there are fundamental differences between individuals with autism and an intra-personal form which suggests that no one factor is able to explain all features of autism within a given individual. What is apparent in the case of autism is that scientists put uncertainty and indeterminacy into discussion with one another and, rather than a well-policed epistemic-ontic boundary, there is a movement between, and an entwinement of, the two. Understanding scientists’ dialogue concerning uncertainty and indeterminacy is of importance for understanding autism and autistic heterogeneity but also for understanding uncertainty and ‘uncertainty work’ within science more generally.

## Introduction

Autism was first described by Kanner (1943) and consists of a dyad of socio-communicative impairments and restricted interests and repetitive behaviours (American Psychiatric Association, 2013, p. 50). Over the last 30 years autism has become:

… the condition of fascination of the moment, occupying a number of cultural locations that reflect a spectrum of wonder and nervousness—the allure of potentially unquantifiable human difference and the nightmare of not somehow being “fully” human. (Murray, 2008, p. 5)

Occupying such a position, autism has attracted a significant amount of attention, not only from the natural and medical sciences but also the creative industries (autism fiction now being a recognised genre), the humanities, and the social sciences.

With regard to the social sciences in particular, I suggest that there are at least three reasons why autism holds a particular interest. First, autism has been described as *the* key diagnosis of the late twentieth and early twenty-first centuries (Nadesan, 2005, p. 3), a ‘living experiment in concept formation of a sort that does not come more than once in a dozen lifetimes’ (Hacking, 2009, p. 506). Second, that living experiment has quite clearly been shaped by a range of factors that are taken to be archetypical of much contemporary scientific practice; factors as diverse as increased surveillance over childhood (Rose, 1985, p. 176; Armstrong, 1995, p. 396; Béhague and Lézé, 2015), deinstitutionalisation (Eyal *et al*., 2010; Evans, 2013), contemporary disciplines such as molecular genetics (Navon, 2011; Navon and Eyal, 2014) and neuroscience (Fitzgerald, 2013; Hollin and Pilnick, 2015), and the experience and expertise of lay groups including parents and (self-)advocates (Silverman, 2012; Hart, 2014). Third, and this is the focus of the current article, despite significant levels of investment (Pellicano *et al*., 2013) autism science is remarkable because it is just so uncertain.

It is well recognised, for example, that during the 1990s there was an ‘epidemic’ of autism diagnoses (Eyal *et al*., 2010, p. 2) with prevalence rates increasing from around 4:10,000 in 1978 (Wing and Potter, 2002, p. 151) to approximately 1:100 in 2009 (Baron-Cohen *et al*., 2009, p. 500). Yet there is little agreement on the cause of this increase and assortative mating (Baron-Cohen, 2006), diagnostic substitution (Bishop *et al*., 2008), and environmental risk factors (Weintraub, 2011) have all been proposed as possible factors. Similarly lacking in explanation is the 4:1 male-to-female ratio in diagnoses (Fombonne, 2009) a finding which, again, has been attributed to basic biology (Baron-Cohen *et al*., 2011) and more complex social factors (Cheslack-Postava and Jordan-Young, 2011).

Most obviously, however, this claim of ubiquitous uncertainty in autism relates to the fact that not only is there no cure for autism, but also that the condition’s cause and manifestation remain hotly contested. To give a flavour; there is significant disagreement over the links between autism, intellectual disability, attention deficit hyperactivity disorder, and other forms of psychiatric diagnosis (Skuse, 2007; Ronald and Hoekstra, 2011); whether traits associated with autism are found in the general population (Bailey *et al*., 1998; Frazier *et al*., 2010); and as to whether autism should be understood as a form of psychopathology at all (Kapp *et al*., 2013).

Perhaps most strikingly, there is an uncertainty as to whether autism symptoms have a common cause or whether they are ‘fractionable’ (Constantino *et al*., 2004; Happé and Ronald, 2008). It is now widely suggested within the scientific literature that it is ‘time to give up on a single explanation for autism’ (Happé *et al*., 2006) and that autism should be characterised as a condition of ‘the idiosyncratic brain’ (Hahamy *et al*., 2015). This is a debate related to the idea of *autistic heterogeneity* and denotes the claim that there may be no one thing which unites all individuals diagnosed with autism (what I will call inter-personal heterogeneity) and that there may be no one thing that explains all symptoms within a particular individual (here called intra-personal heterogeneity).

Drawing upon interview data, in this article I ask two questions relating to these matters. First, how do scientists understand, and then cope with, uncertainties when studying autism? Second, what is the relationship between these uncertainties and claims made about autistic heterogeneity? A core argument is that discussions of scientific uncertainty and autistic heterogeneity are entangled with each other. The indeterminacy of autism shapes scientific talk and practice and is used by scientists to explain the uncertain state of autism science (I call this ‘indeterminacy work’). Simultaneously, I suggest, uncertain science has legitimated and stabilised a heterogeneous autism. Thus, epistemology and ontology can be understood to bleed into, and diffuse through, one another.

## Analytical Perspective

An increasing body of social scientific literature has sought to understand the role of uncertainty in science, detailing the undertaking of ‘practical uncertainty work’ which allows research to remain ‘doable’ by disarming, displacing, and deflating uncertainty (Webster and Eriksson, 2008; Moreira *et al*., 2009; Pickersgill, 2014; Gardner *et al*., 2015). For example, Star highlights (1985, p. 406) four forms of uncertainty—taxonomic, diagnostic, political, and technical—encountered by nineteenth century neurologists and details numerous forms of work which allows research to continue in the face of these difficulties. One of Star’s central arguments is that this uncertainty work—and Star explicitly bases her analysis within the sociology of work—is undertaken in order to transform numerous ‘local uncertainties’ which plague every laboratory into ‘global certainties’ which are presented for a wider audience. Importantly, Star’s ‘wider audience’ is largely a scientific, rather than public, audience. Thus, Star’s analysis of uncertainty stands in contrast to analyses of uncertainty in public, controversial fields such as genetically modified crops (Levidow, 2001, p. 868) and climate change (Demeritt, 2006, p. 463) wherein scientists may be required to adopt an ‘argumentative stance’. In controversial fields such as these it may be that ‘the issue of uncertainty is a strategic element of argument rather than something which causes argument’ (Campbell, 1985, p. 447). This less obviously so for Star, who stresses that the uncertainty work undertaken in her case is not deliberately manipulative as much as it is a mundane and ‘inextricable part of scientific work organization’ (Star, 1985, p. 415).

There are thus important differences here between the work of Star and Shackley and Wynne (1996) who examine uncertainty work in a policy context and focus upon the capacity of uncertainty work to act as a ‘boundary ordering device’ which reconciles uncertainty and scientific authority. While the current case bears more resemblance to Star’s—firstly, because autistic heterogeneity is not a significant policy issue and, secondly, because the stakes during qualitative interview, wherein these data were collected, are lower than in policy debate—there are nonetheless important and relevant methods for reducing uncertainty in science noted by Shackley and Wynne. First, scientists may attempt to quell discussion of uncertainty by promising to reduce it in the future. Second, there may be an attempt made to clarify and manage uncertainty by, for example, containing uncertainty within error bars or circumscribing uncertainty within a narrow academic field (see also; Decoteau and Underman, 2015, p. 473). Third, a condensation of uncertainty may take place so that one form of uncertainty is recognised while another is systematically ignored (see also; Edwards, 1999, p. 462). Fourth:

If the “responsibility” for a major unresolved uncertainty can be firmly located within another discipline, policy domain, or social world, then the work of reducing uncertainty can effectively be displaced (cf. Pinch, 1981) and the uncertainty ceases to threaten the authority of the scientific community. (Shackley and Wynne, 1996, p. 290)

Thus, there may be a displacement of uncertainty into other disciplines (see also; Pinch, 1981, p. 155).

As discussed above, scholars have stressed that on occasion scientists seek not to disarm or deflate uncertainty but, rather, to actively exploit it; uncertainties ‘can be, and have been, employed as political resources’ as Paul Edwards notes (1999, p. 439). Mellor (2010), for example, discusses the public management of uncertainty in relation to the possibility of an asteroid strike and suggests that on occasion uncertainty is promoted by scientists in order to procure research funds. Similarly, McGoey calls attention to the ‘generative and performative nature of uncertainty’, claiming that when ‘a situation is uncertain, it demands attention, debate, funding, and most crucially, experts to determine how the situation should be resolved’ (McGoey, 2009, p. 155). Uncertainty may, then, be used strategically to maintain authority, legitimacy, and research funding (Shackley and Wynne, 1996, p. 280, 282; Zehr, 2000, p. 98; Moreira *et al*., 2009, p. 675).

It should be clear from this discussion that the term ‘uncertainty work’ refers to a wide range of practices and does not necessarily imply any deliberate obfuscation. Certainly, some of the literature cited above seems to detail fairly straightforward instances of what Peter Galison calls an ‘antiepistemology’ where attempts are made to ensure that politically problematic uncertainties are ‘covered and obscured’ by those acting in the ‘art of nontransmission’ (2004, p. 237). Such intentionality is certainly not claimed in other instances (e.g. Demeritt, 2001, p. 347), and instead what is detailed are honest attempts to make work ‘doable’ (Fujimura, 1987). What this literature does insist upon is that uncertainty and work undertaken to reduce, manage, or understand it can be performative and generative; of authority, funds, objects of investigation or, of course, certainty. This generative potential of uncertainty is particularly evident in discussions of uncertainty in autism and the human sciences.

### Uncertainty and Autism

There is good evidence that uncertainty, and uncertainty work, plays an important role in both autism research and in other fields concerned with psychopathology. Fitzgerald, for example, interviewed a number of psychiatrists and psychologists who were concerned with autism and utilised various neuroscientific methodologies in the course of their investigations. During these encounters Fitzgerald describes scientists who have ‘not only a sense of hope, but also a much more ambivalent and uncertain attitude to the future of autism neuroscience’ (Fitzgerald, 2014, p. 256) and, in language reminiscent of McGoey, claims that ‘“low expectation” is a generative force within bioscience … allowing neuroscientists to enact and sustain projects that have an in-built ambiguity or uncertainty’(Fitzgerald, 2014, pp. 258–259).

While in many senses quite different to Fitzgerald’s work, Decoteau and Underman (2015) also stress the generative potential of uncertainty and uncertainty work in relation to autism. Decoteau and Underman examine a high profile legal case in the United States wherein plaintiffs—led by individuals who may commonly be understood as ‘heterodox’ or ‘fringe’ scientists (Collins, 2014)—‘painted the field of autism science as wholly uncertainty’ (Decoteau and Underman, 2015, p. 473). These individuals argued that it was entirely plausible that the administration of vaccines caused at least some instances of autism. High profile, orthodox, respondents with significant ‘scientific authority capital’ (Decoteau and Underman, 2015, p. 480), however, sought to ‘circumscribe uncertainty surrounding autism in order to refocus attention on the potentialities of the genome’. Such strategies, the authors argue, were successful and ‘played a role in clinching and shoring up scientific hegemony on broader issues in autism causation and in steering research development’ (Decoteau and Underman, 2015, p. 473). Regardless of the merits of specific claims made by Decoteau and Underman concerning the evidence given by various respondents (e.g. Decoteau and Underman, 2015, p. 491), the authors certainly detail a clear example of the clarification and management of uncertainty (Shackley and Wynne, 1996, p. 281) in autism and argue for the performative potential of such uncertainty work.

Striking a cord with much of the above work which emphasises the performative potential of scientists’ uncertainty work, it is particularly noteworthy that research which has considered uncertainty within the human sciences has shown that epistemological uncertainties can manifest themselves in ontological realities. Discussing Mild Cognitive Impairment (MCI), for example, Moreira *et al*., state that:

prodromal dementia categories were positioned as possible re-articulations between different laboratories –molecular biology, neuropathology, neuroimaging, and so forth –and the clinic, in an attempt to ‘cool down’ or stabilize some of the uncertainties [in the field]. (2009, p. 671)

The suggestion here, therefore, is that MCI has emerged as a placeholder and as part of an attempt to ‘cool down’ interdisciplinary tensions, dispute, and uncertainties. The very nature of MCI, therefore, may be radically shaped by broader uncertainties within the sciences of dementia. Similarly Pickersgill suggests that epistemic and ontological uncertainties can be off-set with reference to one another, ultimately ensuring that ‘[diagnostic tools] and the psychopathologies they purport to identify become tightly bound together, co-producing the epistemological and ontological coherence of both mental health categories and their diagnostic criteria’ (Pickersgill, 2011, p. 84).

In this article, and drawing upon interview data with leading psychologists and neuroscientists researching autism, I build upon the above literature and its consideration of the performative potential of uncertainty in the human sciences. I show, firstly, that during interviews autism scientists openly discuss various forms of uncertainty. However I also argue that when attempting to account for the recalcitrance of this uncertainty researchers engage in discussion of ‘autistic heterogeneity’, a concept which locates uncertainty within autism itself and suggests that autism is, by nature, indeterminate. Following others (e.g. Hacking, 1995, p. 234; Barad, 2007, pp. 115, 265) I am here differentiating uncertainty and indeterminacy in the following the manner: The term ‘uncertainty’ denotes epistemological claims like ‘I am *unsure* if autism is a real thing’. The term indeterminacy, by contrast, is used to mark claims like ‘I am *sure* that autism is multifaceted’. I argue here that these terms are entwined in autism research for, while a significant amount of methodological and conceptual uncertainty is discussed, reference to autistic heterogeneity also marks a certain claim that autism is a condition determined by its indeterminacy.

In this article, among the first to systematically interrogate the core concept of autistic heterogeneity, I seek to show that autistic heterogeneity is a far from stable concept and is intimately related to a particularly striking form of uncertainty work wherein scientific uncertainties are taken to be inherent in the nature of autism. I conclude the article by thinking about the consequences of this particular understanding of autism.

### Uncertainty and the Importance of Research Context

As is evident from the above discussion, one re-occurring finding in relation to uncertainty is that audience and context matters. The testimonies of experts analysed by Decoteau and Underman suggest that autism is a consistent diagnosis with qualitatively distinct abnormalities (2015, p. 488). By contrast, the talk of neuroscientists interviewed by Fitzgerald is marked by the consistent presence of uncertainty and ambivalence. Autism appears to be, then, more than one even if it is ultimately less than many (Mol, 2002, p. 55).

Method and space is crucial to understanding these differences. Pinch, in a study examining the negotiation of uncertainty in solar neutrino science, states that when considering what scientists say:

one constraint is the potential audience for comments on certainty … when scientists perceive a possible public audience they tend to act defensively and stress the certainty of their own areas—while, at the same time, doubting the certainty of others. (1981, p. 155)

Others have supported this conclusion suggesting that, while scientists may indeed discuss uncertainty publically, there is an attempt to manage its nature in public forums (Zehr, 2000; Moreira *et al*., 2009; Mellor, 2010; Hollin and Pearce, 2015). This conclusion may go some way to explaining the certainty of the testimony examined by Decoteau and Underman which is, in some ways, at odds of with so many other statements of uncertainty in autism research. Previous research, drawing on Pinch, suggests that when researchers are placed into an adversarial, ‘argumentative stance’, ‘there is a strong tendency for critics to accentuate uncertainty and for defenders to claim more solid knowledge’ (Campbell, 1985, p. 448). The courtroom, then, may engender itself to particular statements that would be less readily found in other settings.

With regard to interview methods, Pinch considers it probable that he and his readership are considered a public audience and, thus, that the scientists he spoke to during interviews acted defensively during questioning. Fitzgerald reaches quite different conclusions, arguing that his interviews reflect ‘mundane and private reflections of neuroscientists who are working to understand and articulate their own scientific practice, in the midst of more or less low stakes and anonymous conversation’ (2014, p. 253). Thus, Fitzgerald concludes that:

I do not see ambiguity in this piece as a rhetorical strategy that papers over the cracks. Nor do I see it as an attempt to co-opt opponents who (rightly) worry about where research is going. I see it as a way to think seriously about the complex biosocial space of autism diagnosis and research. I interpret it, finally, as an honest attempt to think, work and live through the sheer complexity of this emerging and growing field. (Fitzgerald, 2014, p. 258)

I am reluctant to pick between these two positions at the outset and rather suspect that both contain some truth, that relations during the interviews I conducted were ‘mobile, reversible, and unstable’ (Foucault, 1997, p. 292). Nonetheless, it is absolutely essential to give a sense of the project in hand in order to better understand the data.

### Context of the Present Study

While methodological details of this project have been published in detail elsewhere (Hollin and Pilnick, 2015, p. 280) several aspects of the study are particularly important in the present context. First, neither uncertainty nor heterogeneity were issues of a priori concern for this project, although the fact that there are a plethora of viewpoints and competing claims surrounding autism was a determining factor in electing to study the condition. This was also a rationale behind the use of qualitative interview as a tool to elicit these wide-ranging views. Second, while the project was designed to be sociological in nature, my first and Masters degrees were obtained in psychology. At the time of the interviews I was also being co-supervised for my Ph.D. studies by an autism neuroscientist known to most, if not all, of the participants. This information was not withheld from participants and was, on occasion, a topic of discussion. As noted, existing research has argued that prospective audience is an important determining factor in discussions of uncertainty and, while it is debatable as to whether my positioning makes these conversations more or less public, that position is worthy of notation even if we are left to speculate on the precise consequences.

The extracts presented here have been anonymised and conclude with a note of both the interviewee’s academic position and the interview number (e.g. Professor, interview 9) although analysis revealed no evidence of systematic differences between academics of different levels of seniority. Some extracts also include my own dialogue and those sections are preceded by an ‘I:’. The interviewee’s response during these interactional extracts is preceded by an acronym based upon their academic position (e.g. PD for Postdoctoral Researcher).

The subsections into which I have divided the data, below, emerged during analysis and each concern a form of uncertainty, or uncertainty work, discussed during the interviews. These sections do not mark pre-existing categories as understood by my interviewees. Interviewees did not discuss these topics separately from one another and I do not wish to suggest, through the creation of these thematic subsections, that they *should* be understood as being entirely independent. The purpose of pulling part these threads in the analysis is so that one might be able to trace those threads back and examine their interdependent and tightly knotted existence in the discourse of these researchers.

## Epistemic Uncertainty and a Higgledy-Piggled Science

The first discussion of uncertainty within the research interviews pertains to a form of uncertainty which arises from an attempt to integrate numerous, diverse, scientific methods when studying autism. As discussed above, these questions relate to what Pickersgill has called ‘epistemic uncertainty’ and, broadly, what Star named ‘technical uncertainty’, where uncertainty has ‘developed as a result of inadequate tools or ambiguous information about techniques’ (Star, 1985, p. 403).

It is certainly true that the number of cognitive tests used to explore autism has exploded and that interviewees in the current study suggested that the sheer range of tests, and the differences between them, lead to contradictory findings and a significant degree of epistemic uncertainty. A Research Fellow, for example, states that:

… I mean the experiments that are used are hugely mixed, erm, the samples that are used varies, the mental state that you’re looking at varies, whether you look at something like a belief versus an intention. So it’s really not surprising that there’s not a huge amount of agreement. (Research Fellow, interview 4)

According to this researcher, uncertainty arises because scientists are examining different ‘mental states’, such as ‘belief’ and ‘intention’, and doing so through a ‘hugely mixed’ set of experimental paradigms; these diverse paradigms, it is reported, yield wildly different results.

A similar claim is made by an Associate Professor who states that ‘ … you look at other areas and it becomes much more higgledy-piggled what people have used, and the task they’ve used and how they’ve measured it’ (Associate Professor, interview 11). Two things are of note in this extract. The first is that, as in the previous extract, which identified an epistemic uncertainty within the vast array of scientific practices concerned with autism, there is an assertion here that cognitive psychology has become ‘much more higgledy-piggled’ and it is this ‘hugely mixed’ set of experimental paradigms which ensure a lack of certainty. A second feature of this extract is that there is a very obvious piece of ‘uncertainty work’ in that there is a displacement of uncertainty into ‘other areas’ and ‘people’, meaning other researchers (Pinch, 1981; Shackley and Wynne, 1996). It is not the Associate Professor’s own work from which uncertainty flows but allied fields; one might imagine that this displacement makes the Associate Professor’s own research ‘doable’ as internal integrity is preserved.

It is not hard to find support for the claim, made by both of these interviewees, that experimental diversity has increased significantly in recent decades. This increasing diversity has been particularly marked following the introduction of a vast range of biologically and neurologically based techniques during the 1990s, the so-called ‘decade of the brain’ (Elam, 2015, p. 47). The claim within these interviews was that the introduction of these new methods has led to conflicting findings and a struggle to reconcile work between forms of analysis. These struggles and anxieties are captured in the following metaphor, where a Professor discusses attempts to integrate neuroscience with experimental psychology:

I mean I think there’s a kind of cultural, erm, narrative that says somehow or other if we can nail it to bits of the brain that we understand it better, er, but (exhales). Er, in fact the nails are not very tight, or deeply driven in, er, they’re, they’re more speculative nails. (Professor, interview 7)

The inability to drive nails between different levels of analysis, in this instance between cognitive psychology and neuroscience, may thus give the impression of heterogeneity and this is a failing of psychology’s ‘cultural narrative’, the assumption that biological levels necessarily ensure that ‘we understand it better’.

It needs to be noted that this Professor is not a significant user of neuroscientific methods and we can thus posit that a particular form of uncertainty work is being engaged in. As noted in the analytic perspective, Shackley and Wynne (1996, p. 290) argue that one form uncertainty work involves attributing responsibility for uncertainty to other disciplines or social worlds. This description seems applicable to several of the extracts that have been presented here in relation to the epistemic uncertainties experienced during research into autism,

This conclusion appears to be at odds with that reached by Fitzgerald (2014), as discussed in the analytic perspective. Fitzgerald reports upon the uncertainties discussed by autism neuroscientists and, while he does not seek to deploy a thematic analysis akin to that being used here, most of the uncertainties discussed would be considered epistemic in nature. Fitzgerald, firstly, dismisses the possibility that discussions of uncertainty could be part of any ‘rhetorical strategy’ (Fitzgerald, 2014, p. 258) and, secondly, suggests that neuroscientists report ‘ambivalence and uncertainty about their *own* practice’ (Fitzgerald, 2014, p. 244, italics mine). In contrast, the evidence presented here suggests that, firstly, those researching autism are likely to displace uncertainty within other arenas and, secondly, that such strategies may well be made in order to make their own work doable by preserving the scientific integrity of its foundations.

## Ontological Uncertainty Over ‘The Autisms’

The second discussion of uncertainty within the research interviews concerns an uncertainty as to whether autism is a unitary ontological entity. This is a discussion based around the classification of autism and the reliability and validity of that classification. To return to the distinction introduced previously, this uncertainty regarding the classification of autism is akin to what Star has called ‘taxonomic uncertainty’ (Star, 1985, p. 397) and Pickersgill ‘ontological uncertainty’ (Pickersgill, 2011, p. 74). Ontological uncertainty reflects a lack of confidence that the label of autism denotes a homogenous, discrete population.

That the Diagnostic and Statistical Manual of Mental Disorders, Fifth Edition (DSM-5) constructs psychiatric disorders as categorical rather than dimensional in nature has, of course, been an issue of intense debate (e.g. Anckarsäter, 2010) and this is a discussion from which autism has certainly not been exempt (e.g. Skuse, 2012). Indeed, the idea that there is ‘no such thing as autism’, and that disparate subgroups of quite different individuals were being lumped together was a frequent claim within the research interviews. A Reader, for example, states that ‘I don’t think there is one autism so to say what, what autism is, is I think a bit of a (.) non-starter to be honest’ (Reader, interview 1). The same point is made by a Research Fellow:

I: … if I was going to ask you what you think autism is, erm, how do you think it’s best described?

RF: (.) Erm. I honestly think that it’s, at the moment, what we call autism is just a collection of many different subgroups. I don’t know, maybe this is controversial, I don’t even know if there is such a thing. (Research Fellow, interview 12)

The lack of certainty in this interviewee’s answer seems evident. Nonetheless, the argument being advanced here, and as with the Reader previously quoted, is that it is unlikely that there is ‘such a thing’ as autism; a faulty prior classification has led to a number of diverse groups of individuals being placed together.

The uncertain nature of autism as a classification is also discussed by a further Research Fellow who states that ‘we’ve got this wonderful “the autisms” term that everybody’s talking about and, no it’s the current very in thing’ (Research Fellow, interview 5). It thus seems conceivable that a conceptual shift and reorientation, whereby unity was sought at the genetic rather than behavioural or cognitive level, could be necessary to reduce uncertainty in the field.

Discussion of ontological uncertainty in autism seems to be very much tied to the context within which discussion takes place. As discussed in the above analytic perspective, in legal settings—where scientists have previously been reported to present ‘more solid knowledge’ (Campbell, 1985, p. 448)—Decoteau and Underman (2015, p. 488) find scientists who confidently describe autism as a consistent diagnosis marked by qualitatively distinct abnormalities. Fitzgerald, by comparison, interviewed researchers who show a marked amount of ‘ambivalence and uncertainty’ about their own work (Fitzgerald, 2014, p. 244). That the current thematic analysis arose from data emerging from within interviews is, then, important. Nonetheless, discussions of ontological uncertainty in autism are not restricted to ‘corridor talk’ and have, in fact, been formalised with discourse (Rabinow, 1996, p. 50). The reference to ‘autisms’ made by the Research Fellow in the above extract, for example, is directed towards a body of work made popular by molecular geneticist Daniel Geschwind (e.g. Geschwind and Levitt, 2007) which suggests that there are numerous, distinct populations currently being labelled as autistic.

## From Uncertainty to Indeterminacy

That discussions concerning uncertainty in autism are so widespread suggests that the topic may be treated in an interesting way within autism science. Nonetheless, the forms of uncertainty identified thus far are broadly similar to those in other scientific domains; we see forms of uncertainty akin to those discussed by Shackley and Wynne, Star, Pickersgill, and Pinch, as well as attempts to deal with those uncertainties which, again, are recognised within the literature. What is particularly interesting about the case of autism, however, is that alongside these recognised forms of uncertainty and uncertainty work there is a further narrative—that of autistic heterogeneity—wherein chance and uncertainty are inherent to the autistic condition itself. This is a claim that autism is an indeterminate entity.

To return to a distinction discussed above, I have used the term uncertainty to refer to claims such as ‘I am *unsure* if results from various techniques are comparable’ (epistemic uncertainty) and ‘I am *unsure* if individuals with autism make up a single population’ (ontological uncertainty). The term indeterminacy, however, refers to statements like ‘I am *sure* that autism is multifaceted’ which reflects a belief about the state of the world rather than ways of knowing it.

A consideration of indeterminacy is of importance in its own right for, both within the interviews and the literature, it is one of the central topics of concern within contemporary autism research. A detailed, critical, reading of the concept has yet to be conducted, however, and the analysis here may allow a better ‘vantage point’ (Pickersgill, 2011, p. 84) from which to comprehend the phenomenon. Moreover, an analysis of autistic heterogeneity is also important because, I suggest, the indeterminacy of autism is used by scientists to explain why it is that autism research continues to be marked by the uncertainties and inconsistencies discussed above. This ‘indeterminacy work’ constitutes a form of uncertainty work; scientific uncertainties are understood as being part of autism itself and scientific work thus remains ‘doable’. This displacement of uncertainty is an example of the entanglement of autism’s nature with its own investigation; uncertain science is intimately related to an indeterminate autism. I use the following sections to juxtapose the foregoing discussions of epistemic and ontological uncertainty with the various and diverse descriptions of indeterminacy provided by scientists during the interviews and subsequently demarcated during analysis.

## Indeterminacy and Autistic Heterogeneity

The notion of autistic heterogeneity, which has at its centre the claim that autism is fundamentally indeterminate, was drawn on repeatedly throughout the interviews and described as one of the most significant features of autism as it is currently understood. As one of the interviewees claimed, autism is best described as: ‘ … a multi-faceted thing that is quite hard to describe with, based on just one core, one variable’ (Postdoctoral Researcher, interview 8). This view is echoed by a further interviewee who thinks that the search for a grand theory of everything is unlikely to be successful:

Well there is no one (.) model like the physicists have, there is no, er, there is no one model that explains er, everything. Er, and I don’t think that we’ll get one model that explains everything … (Assistant Professor, interview 6)

This interviewee, who has a background in the natural sciences, makes a bold claim. Unlike the objects of physics there is no unitary essence to autism, ‘there is no one model that explains everything’. It might be possible for one scientific vision to capture completely the essence of atoms but something in the nature of autism resists such classification. It can immediately be seen how these statements differ from those offered in the previous sections—the uncertainty here does not concern knowledge about autism, the uncertainty is inherent in autism itself.

Interestingly, knowledge about autistic heterogeneity is described as increasing over time—this is an uncertainty about which scientists are increasingly certain. In the extract below a further interviewee, an Associate Professor, explains how they are currently teaching undergraduates about the history of autism research and how there had previously been a search for a ‘holy grail’ (Associate Professor, interview 11) in psychology, one cognitive theory that would explain all the behavioural manifestations of autism. This, it is claimed, is a search which has been largely abandoned:

I: So, (.) you do, you, so it’s not the case, you do think that there’s, there isn’t a holy grail out there, do you think? It’s, it’s not.

L: Oh, I don’t think we’re going to, well, it’s just my personal opinion, I don’t, you know, I don’t think there’s going to be one neat cognitive explanation. (Associate Professor, interview 11)

While the interviewee is careful to frame their answer as ‘just their opinion’ it is noticeable how closely the statement mirrors that of the Assistant Professor above; that autism is always going to resist ‘one neat explanation’, at least at the cognitive level.

This was a repeated claim within the interviews; ‘ … I mean autism is a very heterogeneous condition, okay? I mean I think that’s pretty well accepted now, it didn’t used to be … ’ (Professor, interview 13). Indeed, it is again important to note, and as expressed in the words of a further Professor, that not only has the search for the unitary core of autism at the cognitive or neurological level failed up until this point, in all likelihood future attempts will also be futile:

And there are clearly going to be, well there must be many, many different causes in different individuals on the autism spectrum. Erm, whether those will unify down to a final common pathway in terms of some brain systems or some (.) interrupted neurodevelopmental processes (.), erm, again I wouldn’, hmm, I don’t know how much money I would bet on that ((laughs)). (Professor, interview 18)

Within these extracts, coming from researchers spanning various institutions and levels of seniority, we can see heterogeneity being positioned as an essential aspect of autism itself, a condition with a fundamentally indeterminate nature. These utterances should not necessarily be understood as part of a ‘sociology of low expectations’ (Fitzgerald, 2014; Broer and Pickersgill, 2015; Gardner *et al*., 2015) as researchers’ utterances are neither future-oriented nor do the claims appear to be particularly ambivalent. Instead, in the above extracts one can find the claim that the certainty of science concerning autistic heterogeneity is increasing: the search for the holy grail has been abandoned not because scientists have accepted defeat but because they have come to understand the true, indeterminate nature of autism. It ‘didn’t used to be’ accepted that autism is heterogeneous, but now it is.

There is thus an interesting paradox at the heart of autism research. In the previous sections we saw researchers describe an *increasing* number of uncertainties in the field—increases attributed, in part, to a proliferation of experimental and diagnostic techniques in the field. Simultaneously, autistic heterogeneity has increased—there used to be a belief that there was one explanation for autism but there no longer is. One might, therefore, expect heterogeneity to be understood as some form of experimental artefact, a direct consequence of increased uncertainty. This, however, is not the case; while researchers do acknowledge that new methods have led a proliferation of uncertainty, when it comes to heterogeneity there is a *decrease* in uncertainty. Heterogeneity, it seems, is one of the few certain areas of autism science.

## Intra- and Inter-Personal Heterogeneity

Interestingly, within these discussions of the heterogeneity of autism, it is possible to discern a further sub-division, with two separate types of heterogeneity able to be teased apart during analysis. It is sometimes suggested that there is no common bond between all individuals diagnosed with autism, that these people share no common trait and that there is *inter-individual* heterogeneity present. Simultaneously, it is sometimes claimed that one theory may not be able to explain all of a given individual’s symptoms; this is a model of *intra-individual* heterogeneity, whereby more than one theory is required to explain the appearance of autism in any given individual. These two constructions of autism, that it is indeterminate both within and between individuals, are found throughout the interview transcripts.

The concept of an inter-individual heterogeneity refers to the idea that individuals with autism look very different to one another, genetically, neurologically, cognitively, and behaviourally. For example, a Professor, states that:

But it just seems to me that autism is so heterogeneous. You know that, that, that you’ve got, you know, in order to get, you know, you can give a diagnosis of autism using ADOS [Autism Diagnostic Observation Schedule, a core diagnostic instrument] to someone who’s a Professor at Oxford and somebody who sits in the corner and hand flaps. (Professor, interview 20)

In this extract we can see the now familiar claim that autism is heterogeneous. However, we can also see that this heterogeneity is constructed inter-personally, between a ‘Professor at Oxford’ and ‘someone who sits in the corner and hand flaps’. A similar idea, that ‘everybody with autism is completely different to everybody else with autism’ is expressed by a Reader, below:

when I’ve students and, and they come and work with me and say what have you learned about autism they say well (.) all I’ve learned is that everybody with autism is completely different to everybody else with autism ((laughs)). (Reader, interview 1)

These extracts both unambiguously concern themselves with the differences between individuals with autism.

Inter-personal difference may take various forms. Differences may arise because individuals have qualitatively distinct symptoms to one another; the presence or absence of repetitive behaviours or sensory difficulties, for example. Such a situation is described by a Professor who acknowledges that ‘sensory sensitivities’ do ‘aggregate’ with the ‘problems in social reciprocity and communication’ typically found in autism, but who also insists that there are a number of children who are part of the ‘domain of autistic behaviour’ who do not have these problems:

But I mean you do get this, these things (.) they tend to aggregate (.) but you, you, we, we would argue, we, we also see a lot of kids with very typical problems in social reciprocity and communication, erm, who don’t have the restrictive, repetitive behaviours and specific interests and s’, sensory sensitivities and so on, that use, that are, are, p’, p’, part of the, you know, of that domain of autistic behaviour. Erm, (.) so, anyway, whatever’s going on I would suggest it’s got multiple aetiologies … (Professor, interview 13)

Autism may, then, be qualitatively different in different individuals, the result of ‘multiple aetiologies’. Alternatively, inter-personal difference may be theorised as an issue of severity, individuals being on different ‘parts of the spectrum’. Regardless of the particular hypothesis, the defining issue here is of differences between individuals.

By comparison, intra-individual heterogeneity refers to the claim that there are different causes of autism *within* an individual person. For example, it might be claimed that the causative factors behind communication deficits are fundamentally different to the causative factors behind repetitive behaviours. Such a discourse of intra-personal heterogeneity was also prominent within the interviews and is nicely expressed in the following extract. This Assistant Professor has advocated a theory of autism known as the social reward hypothesis (see Chevallier *et al*., 2012 for details) which argues that, rather than being unable to understand other agents, individuals with autism are simply not motivated to do so. The Assistant Professor is asked how such a hypothesis might explain apparently non-social aspects of autism such as restricted interests or repetitive behaviours:

Well (.) the, there is no direct, erm, there is no direct way where the social, er, reward hypothesis will have a prediction for higher or lower social, er, lower repetitive behaviour … it appears that these two aspects of autism may have different aetiologies so they happen to co-exist in various cases but, erm, there wouldn’t be a single phenomenon that could explain both at the same time, erm, (.) from the looks of it, from, from the looks of the factor analyses that have been done … I mean I think it’s, it’s almost a wrong question to ask, whether, say, for example, the enhanced perceptual function model which, er, erm, I, which erm, erm, [Laurent] Mottron, er, and Kate Plaisted-Grant, er, talk about (.) whether, to ask those perceptual based models that whether they can explain the social phenomenon or to ask a social motivation type, a social based model to ask whether they can explain the perceptual, I mean, why do we need a single explanation? We, we don’t. (Assistant Professor, interview 6)

We can see here that, despite also advocating a form of autistic heterogeneity, this form of heterogeneity is quite different to that described in the previous extracts. Here, individual differences are not mentioned. Instead, ‘two aspects of autism’—‘social’ and ‘repetitive’ behaviours—have ‘different aetiologies’ but ‘co-exist’ in certain individuals. Indeed, the study of autism has advanced to the point where the question has to be asked ‘why do we need a single explanation’—the answer is that ‘we don’t’. Exactly the same point is made by a Research Fellow in the extract below. This research fellow suggests that there are ‘two distinct things’ within an individual with autism:

I find it hard to explain all of the social components with non-social components so I wonder whether there’s two distinct things, erm, that have to coincide for you to have autism but they are distinct. (Research Fellow, interview 12)

These findings of intra-personal heterogeneity have led many researchers to reach the conclusion that there is no ‘single psychological deficit’ and that ‘more of a mix’ of theories is required to explain autism. As a second Research Fellow states:

… people are more accepting of the idea that you don’t have a single psychological deficit. So the days of weak central coherence versus executive functioning versus theory of mind seem to be gone, and people are understanding more that there’s much more of a mix. (Research Fellow, interview 4)

Within the present extracts, and in clear contrast to the autism scientists studied by Decoteau and Underman (2015), autism is constructed as being heterogeneous both intra-individual and inter-individual in nature. Such a condensation of heterogeneity from a multi-faceted to a singular concept is consistent with the conclusions of Shackley and Wynne, discussed during the analytic perspective, who suggest that in order to make uncertainties more tractable, there is an (almost certainly unwitting) process of transformation that ‘occurs when different types of uncertainty are represented as faithfully captured by one designation’ (Shackley and Wynne, 1996, p. 283). By understanding autistic heterogeneity as a singular quality of autism, scientists may well glean a clarity that would be impossible if heterogeneity were explicitly recognised in all its complexity.

## Indeterminacy as Uncertainty Work

Thus far I have detailed the ways in which psychologists and neuroscientists describe themselves as facing, in addition to both epistemological and ontological uncertainties, a condition which is indeterminate by its very nature. Autistic heterogeneity is, I have suggested, exogenous to science, intractable, and confronts autism research at every turn. In this final section I will consider the inter-relations between epistemic uncertainty, ontological uncertainty, and indeterminacy. I argue that claims of autism’s indeterminacy can be understood to be, at least on occasion, a form of uncertainty work. Much as others have suggested that scientists sometime work to displace uncertainty, shifting it away from their own field into other disciplines (Pinch, 1981, p. 155; Shackley and Wynne, 1996, p. 290), I suggest here that understanding persisting uncertainties as being attributable to an indeterminate condition may make research doable and failures comprehensible.

One Associate Professor, for example, states that: ‘ … I think one of the things we do know about autism is how heterogeneous it is, I mean it’s just ridiculous’ (Associate Professor, interview 11). According to this Associate Professor it is not the case that the apparent failure of the cognitive sciences (or some allied discipline) to locate the unitary essence of autism is a failing on its part, as suggested by notions of epistemic and ontological uncertainty. Rather, because ‘ … it’s quite hard to tar everybody with the same brush in something that’s so, erm, heterogeneous as autism’ (Postdoctoral Researcher, interview 19), it is in the nature of autism to elude the descriptions of psychologists.

A further Postdoctoral Researcher makes similar claims in the following extract. I asked the researcher why, given the theoretical and empirical resources devoted to the issue, there are so few certainties in relation to what autism ‘is’ and what ‘causes’ it. This Postdoctoral Researcher quickly put questions of uncertainty into discussion with those of indeterminacy:

I: Mm hm, yeah. So, I mean, it’s quite striking I think that, still, er, despite that we have these, clearly very sophisticated, models and some very sophisticated methods as well, that there’s still a lot of disagreement about, erm, what causes autism and what autism is. Erm, what, why do you think that there remains such a degree of disagreement, erm, when we’ve stud’, studying this condition for quite a long time now?

PD: (.) Yeah. (.) Yeah. Well (.). I think one thing about it is the heterogeneity. (Postdoctoral Researcher, interview 9)

Uncertainty and indeterminacy do not, then, stand separate in the reports of working scientists; the indeterminate nature of autism is instead used to explain the presence of epistemic and ontological uncertainty. Repeatedly, when asked why psychological research has so frequently contradicted itself in terms of research findings, it was claimed that ‘heterogeneity’s probably the biggest barrier and the biggest explanation for why it’s messy’ (Associate Professor, interview 11). Here, heterogeneity is not a construct of science but ‘the biggest barrier’ to science. This is made abundantly clear in the following extract from a Research Fellow:

… So, (.) yeah I guess because of all of that heterogeneity (.) that just makes it a much harder disorder to work with. Erm, and harder to get to the core component, whatever level they happen to be at, erm, because of the heterogeneity, it’s really just getting in the way of us advancing so, although we’ve got a huge amount of autism research in the literature now and a huge amount of money’s been pumped into it in the last ten, twenty years, I think we still don’t have enough to really (.) we haven’t gone far enough with it. (Research Fellow, Interview 5)

Again, heterogeneity is ‘getting in the way of us advancing’ and making it ‘harder to get to the core component’ of autism. It is, once again, in the nature of autism to resist scientific investigation. The reason, I suggest, that this can be seen as a form of uncertainty work is that by constructing heterogeneity as a core component of autism, responsibility is shifted from culture and to nature. Indeed, as other research has shown (McGoey, 2009; Mellor, 2010; McGoey, 2012) and as this interview extract hints at, scientists might even be able to leverage more research funding as a result of this uncertainty; harder problems require more resources and ‘we still don’t have enough’.

## Conclusion

This first question asked in this article was: how do scientists understand the uncertainties surrounding autism. Existing work (Star, 1985; Pickersgill, 2011) has shown that ‘uncertainty’ can mean many things and this article finds support for that conclusion, detailing two quite different forms of uncertainty discussed during the interviews. Firstly, *epistemic uncertainty* suggests that a proliferation of methods and techniques have led to disparate results which have proven hard to reconcile. Secondly, *ontological uncertainty* suggests that the label autism may not reflect a discrete, homogenous population. I also showed that scientists, grappling with these uncertainties and a complex clinical entity, engaged in forms of ‘uncertainty work’; strategies which make their research doable in the face of uncertainty (e.g. Pinch, 1981).

In the second portion of the article I addressed a further question concerning the relationship between these aforementioned uncertainties and the argument made by some scientists that autism is *indeterminate*. During the interviews, discussion of indeterminacy centres on the notion of autistic heterogeneity. Autistic heterogeneity refers, variously, to differences between individuals (inter-personal heterogeneity) and the inability to find a common cause for all symptoms within a particular individual (intra-individual heterogeneity). I showed that on occasion scientists put indeterminacy and heterogeneity into discussion with uncertainty, explaining that they are uncertain precisely because autism is indeterminate. Thus, rather than a well policed boundary between the epistemic (uncertainty) on one side and the ontological (indeterminacy) on the other, there is a movement between, and an entwinement of, the two.

I would like to conclude by noting the two contributions of this piece. The first contribution is of utility to those with a broad interest in uncertainty and uncertainty work in science. The existing literature has consistently emphasised that researchers look to their tools and taxonomies as they attempt to understand uncertainty. The corpus has, thus, largely restricted itself to discussions of epistemology (although see Moreira *et al*., 2009; Pickersgill, 2011 for exceptions). This is most evident in those pieces which focus upon antiepistemologies (Galison, 2004) wherein deliberate attempts are made to obscure the true picture. While no such claims of obfuscation are made here, significant support has been found for existing typologies of uncertainty (Star, 1985; Pickersgill, 2011) and uncertainty work (Pinch, 1981; Shackley and Wynne, 1996). This piece has also, however, suggested that ‘indeterminacy work’—which explicitly concerns not tools or typologies but ontology and objects of analysis—may play an important role and that the boundary between uncertainty and indeterminacy is sometimes hard to police. This finding offers a significant potential avenue of future work and suggested that a much fuller discussion of indeterminacy and indeterminacy work is required in order to fully grasp uncertainty in scientific research.

The second contribution, here, builds upon this first point. While, as noted previously, audience and context is crucial to understanding discussion pertaining to uncertainty, it is important to note that this construction of autism as indeterminate goes beyond the interview setting. The claim that it is ‘time to give up on a single explanation for autism’ (Happé *et al*., 2006) is now widespread and it has recently been suggested that autism be characterised by ‘the idiosyncratic brain’ (Hahamy *et al*., 2015). This analysis is among the first to take autistic heterogeneity seriously, to work through what it actually ‘is’ and examine what it may be doing in the talk and practice of scientists. I have argued that the practices of scientists—their experimentation on and theorisation of autistic heterogeneity—have been both performative and productive, having material consequences that have changed the nature of autism. If this is so, if ontology and epistemology cannot be so easily parsed, then all with a stake in autism need to take heterogeneity seriously and to examine its ethical, epistemic, and ontological consequences.
